# Relatives’ experiences with abuse and neglect in Norwegian nursing homes. A qualitative study

**DOI:** 10.1186/s12913-021-06713-9

**Published:** 2021-07-11

**Authors:** Susan Saga, Lene Elisabeth Blekken, Sigrid Nakrem, Astrid Sandmoe

**Affiliations:** 1 Department of Public Health and Nursing, Faculty of Medicine and Health Sciences NTNU, Norwegian University of Science and Technology, Trondheim, Norway; 2 Norwegian Centre for Violence and Traumatic Stress Studies, Oslo, Norway

**Keywords:** Nursing home, Long-term care, Relatives, Next of kin, Elder abuse, Neglect, Qualitative, Interview, Staff-to-resident abuse, Resident-to-resident abuse

## Abstract

**Background:**

Elder abuse in nursing homes (NH) is a widespread and complex problem. Residents’ ability to share their experiences are impeded, due to a high degree of cognitive problems and frailty, and previous studies are thus mainly based on reports from staff. Therefore, we aimed to give voice to the residents by investigating their relatives’ experiences with elder abuse in NH.

**Methods:**

Qualitative individual interviews were conducted with 16 relatives of residents with experience of abuse and/or neglect in NH. Content analysis was used to analyse the data.

**Results:**

Relatives perceived neglect as most pervasive and staff-to-resident psychological abuse as a key problem. Physical abuse was mostly related to resident-to-resident aggression. Relatives perceived elder abuse in NH to be related to low competence among staff, low staffing, poor NH leadership, working cultures characterized by fear and loyalty to employer or co-workers, and a lack of individualized care for the residents. Furthermore, relatives themselves experienced maltreatment from NH, which caused them to suffer stress, anxiety and distrust. Relatives also expressed a need to compensate for lack of care.

**Conclusions:**

Relatives of NH residents who had experienced abuse reported that neglect of basic care and individual rights was predominant and viewed organizational explanations as most important. Relatives perceive themselves as collaborators in care and are emotionally attached to their family member. Therefore, if relatives experience resident abuse or neglect, it inflicts a feeling of being mistreated themselves, particularly if they are not listened to or their notice of abuse on the part of the resident is ignored or trivialized. Including relatives in a committed partnership with NH in care practices is not only a valuable path to reduce the risk of abuse, but it also leads to a more sustainable healthcare with high standards of quality and safety.

## Background

Nursing homes (NH) are settings for long-term medical treatment and nursing care for the frail older persons in our society, and at the same time a home where the residents spend the last phase of their lives. Hence, NH are expected to provide quality care, complying with the human dignity of the resident, and collaboration, honesty, and mutual confidence that ties together families and staff [1]. Still, a growing amount of research has revealed that abuse and neglect frequently occur in NH in many countries [2–10], including Norway [11–13]. The World Health Organization (WHO) recognizes elder abuse and neglect as a global public health problem [14], with a range of serious health consequences, including increased risk of morbidity, hospital admissions, institutionalism, and mortality [15–17], in addition to violations of human rights, dignity and well-being of the older person [15].

The WHO defines elder abuse as: “a single or repeated act, or lack of appropriate action, occurring in any relationship where there is an expectation of trust which causes harm or distress to an older person” ([18], p.3). Five types of abuse are generally recognized: physical, psychological, financial, sexual, and neglect [19]. The type of abuse is further categorized according to the relationship between the key stakeholders, and in NH, it is often divided into staff-to-resident abuse [11, 12, 20], family-to-resident abuse [21], and resident-to-resident aggression [22–24].

### Prevalence of elder abuse in NH

A systematic review and meta-analysis of the prevalence of elder abuse in institutional settings suggested an overall abuse estimate of 64% [20]. This estimate was based on NH staff reports of abuse of patients for 1 year. Prevalence estimates for abuse subtypes as reported by residents themselves were highest for psychological abuse (33%), followed by physical (14%), financial (14%), neglect (12%), and sexual abuse (2%) [20]. A Norwegian cross-sectional study of elder abuse in NH found that among the 3693 nursing staff who participated in the study, 60% reported they had perpetrated one or more incidents of abuse during the past year [12]. Psychological abuse and neglect had the highest prevalence, at 40 and 47% respectively. Physical abuse was reported by nearly 10%, while most staff in this study reported that they had never committed financial or sexual abuse.

NH residents are also exposed to abuse from co-residents. In a prevalence study, 20% of residents had been involved in at least one incident of resident-to-resident aggression during a one-month observation period [22]. In Norway, a survey of staff observing resident-to-resident aggression found that nearly 90% had observed one or more incidents of aggression between co-residents during the past year [13]. Lastly, there is a lack of prevalence studies related to elder abuse committed by family members and/or close friends inside the NH.

### Risk factor or determinants of elder abuse in NH

Descriptions of elder abuse reveal that determinants for abuse and neglect in the NH context are complex and multifactorial [25]. An often-used theoretical model is the ecological model, where determinants of abuse and neglect are divided into five levels: individual, relational, institutional, societal, and the chronosystem, with a dynamic relationship between the coparticipants in contexts at several levels [25]. Factors at each of the five levels can either increase the risk of abuse and or be proactive, thus reducing the risk of vulnerability to abuse.

On an individual level, it is recognized that residents in NH may be particularly vulnerable to abuse and neglect. This vulnerability stems from cognitive impairment, behavioural abnormalities, and/or physical impairments [3]. Studies have reported higher rates of physical abuse in residents with dementia and those with physical impairment [24, 26]. Further, certain staff characteristics are also predisposing for elder abuse in NH at an individual level. Wang et al. [27] found that staff who were younger, less educated, lacking specific training, and perceived a greater burden in their work displayed a tendency towards more abusive behaviours. In addition, stress and burnout have been identified as determinants related to staff characteristics [10, 28, 29].

On a relationship level, staff who experience conflicts with residents, such as managing residents who are unwilling to undress or those who have aggressive behaviour, are more likely to admit to having abused a resident [10]. Other studies have reported a stressful relationship between caregiver and resident to be a determinant for abuse [21, 30, 31].

At an organizational level, studies indicate that staff members working in urban areas are less likely to commit acts of abuse and neglect of an emotional and physical character than staff in rural areas [32]. Low staff-to-resident ratios and high staff turnover have also been found to diminish care quality and are determinants of elder abuse and neglect [29, 33, 34].

Regarding determinants at a societal level, studies and discourses within law, medicine and social science research prevail. Studies have highlighted ageism, the loss of self-determination and perceptions about how ageism affects the healthcare services that are delivered [35, 36]. At the fifth level, the chronosystem, time will impact multiple levels of potential abuse over time and life span, e.g., the impact of length of NH residence on the likelihood of abuse occurring [25]. A qualitative study from Sweden found elder abuse to be related to older persons’ perceptions of their changing roles at the individual level, in the family and in society [37].

### Residents and relatives in NH

In Norway, approximately 39,600 residents, which is approximately 13% of the population over 80 years, live in NH, and mean age of residents is 85 years [38]. Approximately 80% of residents have dementia, and most have significant deficiencies in activities of daily living (ADL). NH have nurses on duty 24 h a day, and the staff comprises registered nurses, licensed practical nurses and unskilled labour. Additionally, an employed physician has the medical responsibility for the NH residents but is only available a few hours a week [39]. In Norway, the municipalities have a statutory obligation to provide NH services to those who need it [40]. Most Norwegian NH are owned and run by the municipalities and financed by taxes and resident payment. However, there are also some private non-profit and for-profit providers [39]. Laws and regulations provide a common legal framework for how NH in Norway are managed and organized, securing a relatively homogenous public service across the country [40]. Consequently, all NH are accounted for and subject to governmental control.

Due to the high degree of cognitive problems in this population, using residents as informants in studies of elder abuse is usually a challenge [21]. An alternative way to give voice to the residents is through next of kin [41]. Generally, relatives know well the life history of their older family members, the way they have lived and how they have maintained their dignity and self-respect. Research indicates that relatives are capable observers of suspected abuse and neglect and are willing to speak quite frankly if they are not linked to any specific institution or area; thus, they may act as effective proxies for older residents [41, 42]. A Swedish study that explored relatives’ perceptions of elder abuse in NH found that abuse was viewed as a violation of an older person’s identity [41]. This was related to staff’s failure to take into consideration the knowledge of relatives regarding the resident’s appearance, daily routines, and preferred activities in daily life within the institution.

Studies of relatives’ involvement in issues of resident dignity, integrity or well-being stress the necessity for staff to build trust and relationships with relatives to ensure that the resident’s voice is considered [41–45]. In a Dutch study of end-of-life care in 34 NH, 252 family members reported unpleasant experiences such as neglect and lack of respect for the patient [46]. In a Canadian study, family members experienced that resident-to-resident abuse was largely normalized by the institutional context it occurred in [47].

None of the studies presented above investigated direct experiences and perception of abuse and neglect in NH experienced by residents or their relatives. Therefore, the study aimed to explore the relatives’ experiences with elder abuse in NH.

## Methods

In the present study, we explored how relatives experience abuse and neglect of residents in NH using a qualitative design. Qualitative methods give insight into human practice, experiences, thoughts, expectations, motives and attitudes, and strengthen our understanding of why people act the way they do [48]. The study is part of a larger study funded by the Research Council of Norway (NFR) (Project Number 262697).

### Setting/sample

We posted information about the study on the social media platforms of collaborative private non-profit organizations, the Norwegian Centre for Violence and Traumatic Stress Studies (NKVT) and the Norwegian University of Science and Technology (NTNU). Relatives contacted the researchers for an interview. The inclusion criteria were close relatives of NH residents who had experienced abuse or neglect and: 1) were or had been close relatives of an older resident in a NH for at least 1 year, and 2) that it was no longer than 5 years since they were close relatives of a resident in a NH. In addition, the snowball method was used in the sense that we asked informants whether they knew of others who fitted the study criteria. The informant then contacted the potential new informant with information about the study and reported back to the researcher if the potential new informant agreed to participate.

The recruitment and data collection were conducted simultaneously during a two-month period. We used a purposive recruitment strategy and included informants that could cover the aims of the study [48]. The inclusion of new informants was based on continual assessments of the themes and conceptual depth that emerged from initial analysis of the interviews. We stopped recruitment of new informants when we considered that saturation was attained in the collected datasets. Thereby establishing an inductive thematic saturation [49]. The final sample consisted of two men and 14 women, a total of 16 relatives (Table 1). Geographically, informants were recruited from all regions of Norway and both urban and rural areas. The informants were ethnic Norwegians and were all working, except two retired informants. The informants worked or had worked in health care, social care, academia/education, design, engineering, public transportation, as leaders, as business office clerk, and as self-employed in business.

**Table 1:** Informant characteristics

Informants *n* = 16	
**Gender** (numbers)	
*Male*	2
*Female*	14
**Age** (Years)	
*Mean (range)*	59 (49–72)
**Relation to nursing home resident** (number)	
*Wife*	2
*Daughter*	12
*Son*	2
**Length of stay in NH for parent/spouse** (years)	
*Mean (range)*	4 (1–10)

### Data collection

Data for this study were collected by individual, semi-structured in-depth interviews [48]. The interviews were carried out in April–June 2020. Due to the COVID-19 situation and sudden lockdown of society in Norway, the interviews were conducted by telephone. All interviews were conducted one-to-one with the informants by SS, LEB and AS. The interviewers are all researchers with PhD’s and have long experience in conducting research interviews. An interview guide with open-ended questions was used (Table 2). The interview guide was piloted in a focus group and enhanced according to their comments. To obtain an information-rich description of the informants’ experiences, a narrative approach was used for the interviews, encouraging the informants to freely talk about their experiences as relatives [48, 50]. During the interview, the interviewer repeated and summarized the expressions of the informants and asked them whether it was correct. The interviews were audio-recorded and transcribed verbatim, retaining frequent repetitions, pauses and emotional expressions. The quotes used in the manuscripts were rewritten into full sentences in order to retain the full meaning of the expressions. Transcripts were reviewed upon completion by the first and second authors to ensure that they reflected the content of the interviews. Each interview lasted for 50–90 min.

**Table 2:** Interview guide

Topic	Key questions
Introduction	Can you describe what you define as abuse and neglect in nursing homes?
Your experiences of abuse and neglect as a relative	Can you describe your experience of abuse and neglect to the resident of which you are a relative?
To detect elder abuse and neglect	Can you describe how you found out about incidences of abuse and neglect in the nursing home?
	Can you describe the communication with staff or nursing home managers?
	Can you describe if there are things that makes it difficult or challenging to report such incidents?
Nursing home management of reported abuse and neglect incidences	Can you describe how the nursing home managed the reported/detected incidences of abuse and neglect?
	Can you describe what is challenging in such a situation?
	Do you dare to be honest when there is something you are not happy with regarding the care of your family member?
The role of relatives to prevent abuse and neglect in nursing homes	Can you describe how you as a relative may contribute to prevent abuse and neglect?
	Can you describe how you want your family member’s needs to be met in the nursing home?
	Can you describe what was important for you to convey about your family member when he or she moved to the nursing home?
	Can you describe how you think your family member experiences the events in the nursing home that you have described?

### Analysis

Graneheim and Lundman’s [51, 52] manifest and latent content analysis was used to analyse the data. The interviews were read in their entirety by the first and second authors to get an overview. The process of looking for meaning and patterns in the data began during the first reading by marking text in the transcripts and writing short keywords. Thereafter, work began to identify units with an independent meaning in relation to the research question. These meaning units were condensed and subsequently coded. The tool MindManager 2020 was used to code the data material and abstract into sub-themes and themes. By moving on to sub-themes and themes, we moved from the manifest to the latent content of the text by describing an interpretation of the underlying meaning [51, 52]. To ensure that the analysis was performed reliably, the coding process was performed by two researchers independently (SS and LEB). During this phase, they met for a critical review and discussion of preliminary codes, sub-themes and themes. Further, three selected transcripts were read by two more researchers (SN and AS) before a joint analysis meeting where coding and preliminary themes were presented and discussed to reach an agreement. The first author then critically assessed codes, sub-themes, themes, and selected quotes to examine whether they provided a representative picture of the material.

### Ethical considerations

Ethical approval for this study was given by the Norwegian Centre for Research Data (NSD), Registration No: 740981. All participants received oral and written information about the study prior to the interview and NSD approved that the participants could give an oral audiotaped consent to participate in the interviews and for the use of the data from the interviews. All identifiable characteristics are excluded from the presentation of data to ensure the anonymity of all individuals.

## Results

The qualitative interviews focusing on NH abuse elicited responses in two overarching areas - resident abuse and maltreatment of relatives (Table 3). Firstly, the informants predictably elicited responses on the theme “resident abuse”. Secondly and more surprisingly, they described how they as relatives felt subjected to maltreatment from the NH when asked about their cooperation with the NH.

**Table 3:** Themes and sub-themes

Theme	Resident abuse	Maltreatment of relatives
**Sub-themes**	Abuse characteristics	Characteristics of relative maltreatment
	Explanations for resident abuse	Consequences of relative maltreatment
	Consequences of resident abuse	

### Resident abuse

In the “resident abuse” theme, three sub-themes were generated: 1) abuse characteristics as perceived by relatives, 2) how relatives explain elder abuse in NH and 3) consequences of elder abuse.

#### Abuse characteristics (Fig. 1)

Relatives expressed that neglect was perceived as the most common form of abuse in NH and described neglect more as a rule than an exception. Relatives reported many instances of inadequate grooming related to both appearance and hygiene, and well-being. Relatives reported that residents were not given baths or showers, their skin was not moisturised, their hair was not properly combed, nor were toenails cut: *‘And when we take off one shoe, it’s full of blood. […] The nails had curled and gone in. It was absolutely terrible’* (informant 13).

**Fig. 1:**
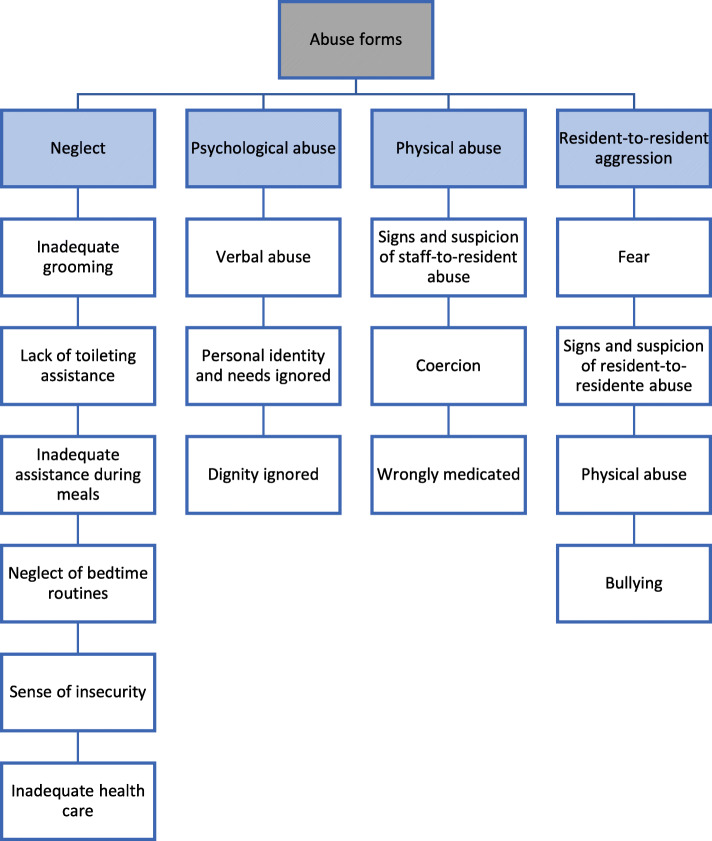
Examples of abuse forms reported by relatives

Relatives reported that residents were not given proper assistance in toileting. Incontinence pads were not changed, even when this was requested by relatives or the residents themselves. One relative described the experience: ‘[Mother]…*had chronic urinary tract infections. And I told them to change her a lot, so she didn’t have to sleep with it. “Oh yes, they would do it,” they said. But I groomed her every day when I came in. And I asked many times: “When did you last change her [diaper]?” Then I came in at five o’clock. No, they hadn’t changed her during that shift. And she was wet every time I changed her*’ (informant 11). The informants also experienced poor dental care to the extent that residents lost their teeth or had problems with eating or talking due to poorly attached dentures.

Relatives also experienced that residents received inadequate assistance during meals: *‘Eventually, I discovered that they didn’t give my mom enough food. If she doesn’t eat fast enough, they take away the plate of food and say: “You know, you’ll get supper afterwards*”’ (informant 14). Informants reported that residents received too little food or were being served food they did not like, despite relatives having repeatedly explained such issues to staff.

The informants also addressed issues regarding bedtime routines and nocturnal sleep patterns, for instance, being put to bed for the night too early: ‘*I came up at six o’clock, and she was put to bed. Then she’ll sleep until the next morning and even longer*’ (informant 7). Several relatives reported similar stories: ‘*We came to her room, but we hadn’t given notice that we were coming. She was lying down. And when we said: “But it’s not even three,” they replied. “Yeah, but if we close the curtain, she doesn’t see the daylight, and she doesn’t understand that it’s daytime”’* (informant 13). Another informant said that the nursing staff turned the resident’s hearing aids off at seven so the resident would become sleepy earlier. Also, they did not always assist the residents up from bed in the morning, especially during weekends when there were fewer staff.

Relatives described problems related to the resident’s sense of security at the NH. Relatives witnessed that nursing staff did not always respond to alarms or cries for help from residents, and it took a long time before they responded: ‘*It takes a very long time when she calls for help*’ (informant 15). Some of the residents were left behind or forgotten by staff or even called relatives at home from their rooms and asked them to notify the NH that they needed help.

Informants also described instances of inadequate medical treatment and follow-up of existing medical conditions, as well as harm or injuries that occurred after admission: ‘*She had clearly been injured and cried a lot due to the pain. [...] I wanted an x-ray examination for her. She didn’t get it; she was refused medical attention. A week after the fall, she got an x-ray, and it turns out that she had two fractures in her pelvis*’ (informant 9).

The informants described experiences of psychological abuse, such as ignoring, yelling, ridiculing and offensive communication with the residents: [staff saying]: ‘*You have to stop fussing. I’ve just been with you. Now I’m so tired of your fuss*’ (informant 6). Moreover, relatives also described “hard reality orientation”. One informant reported, for instance, how her mother would sit in the wheelchair and say: ‘*“Drive me home!” And then they replied: “You have no home”’* (informant 5). The relatives expressed that staff sometimes acted in a way that threatened the residents’ dignity. For instance, informants reported that staff did not knock on the door before entering the resident’s room. Further, residents had to sit with incontinence pads with faeces during meals and together with other residents and their relatives. They also reported that staff spoke loudly and freely about intimate and private subjects in common areas.

In the interviews, informants gave examples where the residents’ identity and personal needs were ignored by staff. For instance, several informants referred to situations where staff did not respect the residents’ belongings, but loaned them out to other residents without asking, with the result that things disappeared. Individual needs, habits and routines were also being ignored. One informant said the staff were frustrated because her mother would not eat some days. The problem was that the staff kept giving the resident porridge, although the relative had been telling the staff for 2 years that her mother did not like porridge. Another informant talked about her mother: ‘*But I had written that list. What she liked and what she didn’t like and all that. It was posted in her room. And I wrote that she doesn’t like red lemonade. You know what ... The day before she died, there’s a glass of red lemonade. Then she’d been there for over a year*’ (informant 11). Relatives explained how they had created clothing strategies for a resident who was blind so she could find what she needed, but the staff did not bother to follow up on it. Residents who had been very particular with their clothes and appearance now appeared uncombed and dressed in random clothes.

The informants also reported physical abuse, including situations where residents were subject to heavy-handed approaches by staff during care, leading to suspected bruising or other injuries. When the relatives detected bruises on the residents, the staff did not always know the reason for it: ‘*... I was talking about the bruises. I don’t know what’s really been going on, but they must have handled her in a hard manner, obviously. There were times when I came where she was going to explain things to me, but she was demented. It wasn’t that easy for her to find the words’* (informant 3). A few relatives reported suspicion of sexual abuse: ‘*I’ve suspected there’s been sexual abuse, but I don’t have any evidence*’ (informant 5).

Relatives also expressed how the use of coercion could lead to physical abuse. Residents were physically restrained or not allowed to walk freely: ‘*I was standing in front of that door to her ward, and I heard her scream. She screams: “Open the door!” I press the button, and the door opens. Then I see a nurse, [...] along with another nurse drag her across the floor. And then I walk over to her, I scream: “What the hell are you doing to my mother?” Right. And then I embrace my mother, my mother [embraced] me. And then, she is calm, and she’s crying on my shoulder*’ (informant 14).

More often, physical abuse of residents was reported as occurring between residents, as these often are residents with dementia: ‘*There are marks on her, so we ask: “Where did those marks come from?” They said: “It’s a resident.” There’s always a resident. But I haven’t seen that other than that I’ve seen that the one who is violent, she goes and rips things out from their hands while they sit there. It’s a pretty rough and big lady*’ (informant 13). The relatives said that residents pushed, beat, kicked or bullied co-residents. They also had examples where their loved ones feared that other residents could come into their room during the night.

Several relatives reported that residents who feared co-residents due to agitation or abusive behaviour were sedated by staff instead of protecting or secluding them: ‘*She has been medicated due to co-residents’ agitation and violent behaviour because it affected her so much that she became afraid’* (informant 10). Informants also reported medical abuse, where residents were given too little pain-relieving medication, with physical pain and withdrawal issues as consequences, in addition to situations of forced medication.

#### Explanations of elder abuse

Even though the relatives were not asked directly why they perceived elder abuse happened in NH, they spontaneously expressed a wish and a need to understand why the abuse occurred.

Informants mentioned low staffing as an important explanation for abuse. For instance, the relatives perceived a lack of staff present in living rooms and corridors to detect residents’ individual needs and avert potentially harmful situations such as agitation and violence: ‘*The largest neglect is that there are not enough people to observe these demented people sitting in the big living room*’ (informant 9). The relatives also expressed that the nursing staff had insufficient expertise, particularly in relation to dementia and how to handle challenges associated with it: ‘*Is the anomaly that she does it or is the anomaly that the ward and the system are not well enough equipped to prevent her from doing it?*’ (informant 8). In addition, there are many unskilled workers among the staff: *‘They pick people up right off the street, with almost no training, and let them start caring for people with this type of disease’* (informant 16). The informants seemed to agree that there was both skilled and unskilled staff who were personally unsuitable for working with frail older residents. However, they also acknowledged that there were staff who cared for the residents and did a great job: ‘*And that’s what I’ve been most pissed off about, that I think there’s so little competence... Not everyone, because there are many wonderful ones here, and I’d like to say that there are so many amazing ones. But there are some cases where you see it is quite frightening, the ones that are there*’ (informant 6).

An explanation that emerged from the interviews, connected to lack of competence, was the general lack of individualized care and adaptation of care practices. For instance, the mother of one of the relatives had asthma, and inadequate medication for her condition led to breathing difficulties, which in turn caused anxiety and agitation. Furthermore, this resident’s agitation also led to anxiety in other residents. Thus, the lack of holistic approaches and the absence of a basic understanding of what caused the resident’s behaviour had negative effects for both the resident and her co-residents.

The relatives also pointed to lack of leadership and other organizational challenges as an explanation for elder abuse in NH. They referred to the absence of adequate resident follow-up by an assigned primary nurse, language challenges among immigrant nursing staff, and that issues reported to the NH were not adequately addressed by unit management. The workplace culture was also highlighted as an important explanation for elder abuse in NH. The relatives talked about staff who would defend themselves or trivialize incidents if they received reports of neglect from relatives. The relatives described a fear culture among staff, who were afraid of sanctions from their co-workers or feared losing their jobs. One of the relatives exemplified this with a talk she had with a former nurse of her family member after the family member had been moved to another NH institution due to a long conflict with the former NH: ‘*And then she said: “You know what, I think about that time at [name of NH] when you were there. I couldn’t help you. If I tried and the others saw that I helped you or supported you ... They were simply afraid of losing their jobs.” […] She works in the school system now, and she said: “I get sick, I get a stomach-ache when I think about these leaders, and I think about how it was there. And I will never ever work in healthcare again”’* (informant 16).

#### Consequences of elder abuse

Relatives pointed to deteriorating health in their family members after admission to NH such as weight loss, teeth falling out, development of bedsores and a vicious cycle where the residents’ ADL functions were gradually taken over by staff. Some relatives even believed they were witnessing some sort of euthanasia: ‘*Active euthanasia is prohibited in Norway, but if one commits a “Sorry, we neglected because we forgot about it”, then what is that? You’re pushing. You push the resident over the doorstep*’ (informant 2). The informants told us that residents felt unsafe when they were alone with co-residents, fearful when they woke up at night with a co-resident in the room, and insecure when they were exposed to yelling and reality orientation from staff for behaviour the residents themselves did not understand nor comprehend.

Relatives also talked about sadness, depression, resignation, or a feeling of being objectified: ‘*My husband [the resident] says that: “Eventually I become completely indifferent, or I will perish*”’ and: ‘*You become a thing. You’re not a human being*’ (informant 1). One relative said her mother wanted a pill to end it all. The mother of another informant called her daughter every day and cried because she wanted to go home, even though she had been at the NH for one and a half years. The relatives expressed that unwanted behaviour from the residents, such as agitation and aggression, was a direct result of neglect and abusive communication from staff.

### Maltreatment of relatives

In the “Maltreatment of relatives” theme, two sub-themes were generated: 1) Characteristics of abuse of relatives, 2) Consequences of abuse of relatives.

#### Characteristics of relative maltreatment

First and foremost, the relatives expressed how their parents’ or spouses’ suffering was also their suffering: ‘*And I have suffered so much with him, that what they did to him, they also did to me*’ (informant 16), and further: ‘*You leave one that ... whether it is a small child or ... a helpless person in the custody of others, and you constantly walk around insecure and fail to rest assured that ... do they get good care, are they taken care of? So, it’s a huge load. At first, it’s hard to have them at home, but at the same time, it’s so hard to leave them to someone because you’re so unsure*’ (informant 16). Many relatives told how they frequently were barred from involvement in the care of their parent or spouse. They were not told about accidents or incidents, were not getting to meet with staff to discuss the resident, and they did not feel they were listened to, or their advice taken, even though they were the ones who knew the resident best.

Relatives told how they repeatedly talked with staff, and sometimes to the care manager, about the care of their parent or spouse, and how they most often were not listened to: ‘*…through the close contact I had as a relative with other relatives in the NH, and also as head of the resident council at two of the city’s NH, I received some information, sent by email, in despair, anger and frustration that nothing was done*’ (informant 2). Relatives experienced direct disregard from staff, and staff who had been told by their care manager not to speak to them after disagreements. They said care managers sometimes denied that incidences reported by relatives had happened, and many relatives experienced rejection and arrogance from staff when these incidences were addressed: ‘*When we enter the NH, I notice that people, the staff that I have such a good relationship with, they give me a dirty look. I just don’t understand anything. They didn’t greet me. I sat with my mom for half an hour, and then I thought: “What’s going on, what’s happening?*”’ (informant 9). Many relatives found it difficult to be assigned the role of “troublesome” relative. They felt it was a struggle to repeatedly ask the staff about their parent or spouse or suggest different approaches towards their parent or spouse. One informant expressed how her feelings of fear for her husband were misinterpreted by the staff: ‘*But when I was despairing, and I was so scared that he was going to die of side effects, then they said to me: “We don’t care that you’re mad. You can just be angry because we’re the ones in charge”’* (informant 16). Relatives experienced that cooperation was on the terms of the NH and that relatives were unwanted: ‘*As relatives ... there’s a hierarchy here. And as a relative, you’re really on the bottom*’ (informant 16).

#### Consequences of relative maltreatment

Relatives expressed a sense of powerlessness regarding the system as a whole – after addressing incidences to staff directly, to care managers and, for many of our informants, also to the County Governor. Relatives experienced that things mostly did not improve; instead, they were left with a feeling of sadness and grief, and experienced stress and anxiety reactions: ‘*And every time I went there at the end, I had to visualize and use exercises to manage to walk in the door. […]. When I was going to enter the NH, my body almost refused to go inside. […] It was terribly unpleasant. It was absolutely horrific*’ (informant 16).

The interviews revealed a pervasive distrust towards how the residents were treated. Consequently, the relatives’ distrust made it difficult to have an open and honest dialogue and cooperation regarding the treatment of residents: ‘*It’s not only the good things you want to bring up, but I had never shared my… my thoughts and feelings towards the staff in any meetings. I wouldn’t have trusted that they embraced and receive it in a proper way*’ (informant 10).

Relatives also reported that they feared giving the staff feedback on negative events due to fear of retaliation. Only a few informants mentioned retaliation from the care staff in the form of worsened care for their loved ones. However, most of our informants expressed fear of giving negative feedback: ‘*And then they will somehow punish Dad. This sounds completely paranoid, but we are afraid that Dad will not get the good care he needs. He can’t speak up about his own needs by himself*’ (informant 4).

Relatives expressed how the NH inadequacy made relatives compensate for lack of care out of fear of what might otherwise happen: ‘*Had to try to compensate for it. Get up and walk with her when we were there, take her out when the weather was right. Make it nice for her… It was painful. There were awfully many times I left with a lump in my throat*’ (informant 12). Relatives said they needed to be the voice for the residents who by themselves were unable to explain how they were doing or demand adequate care. Finally, the relatives expressed a deep concern for residents who did not have relatives to actively support them: ‘*I think if they don’t have relatives, how are they supposed to get help?*’ (informant 13).

## Discussion

This study found that relatives whose loved-one experienced abuse or neglect perceive neglect as most pervasive, and they perceive psychological abuse from staff-to-resident as a key problem. Physical abuse was mostly related to resident-to-resident aggression. Relatives perceived elder abuse to be related to low competence and organizational factors such as low staffing, poor NH leadership, a working culture characterized by fear and loyalty to employer or co-workers, and a lack of individualized care for the residents. The consequences for residents were of a physical, psychological, and existential nature. More unexpectedly, we also found that relatives themselves experienced maltreatment from staff, which caused them stress and anxiety, fear of retaliation from staff towards their parent or spouse, lack of trust, and need to compensate for lack of care.

### Abuse forms

Regarding neglect, we found that relatives whose loved-one’s experienced abuse or neglect expressed neglect as the most common form of abuse in NH, and they describe neglect more as a rule than an exception. This is in accordance with findings from a recent Norwegian prevalence study of neglect and abuse as reported by NH staff [12]. However, in a systematic review of abuse in institutional settings, neglect was less prevalent than psychological abuse, physical abuse and financial abuse [20]. Although our study is not a prevalence study, these patterns seem to contrast with our finding of relatives’ experiences of abuse. Our study explored elder abuse in NH as experienced by people who have themselves witnessed these issues first-hand. The relatives in our study described neglect related to the residents’ grooming, dressing, toileting, meals, bedtime routines, sense of security, and healthcare. Buzgovas’ descriptions of hygiene and healthcare neglect [21] showed the same as our study. Neglect is linked to the concept of “missed care” [53]. Kalisch [54] and Kalisch et al. [55] viewed missed care as a paradoxical relationship between the theory-practice gaps; nurses knew the appropriate standards of care, yet regularly failed to meet adequate and expected standards of care delivery. Certainly, when aspects of care are neglected, this may result in negative outcomes, including fatalities [56].

Our informants experienced that their loved ones were exposed to psychological abuse, such as yelling, being ignored and ridiculed. They perceived that the NH did not adequately preserve the resident’s dignity and identity. This is in accordance with Buzgova [21], who found “rights violation” as an additional category of “psychological abuse”. Dignity is a complex concept that has been linked to identity and associated with respect and autonomy [57]. It can be questioned, however, whether others can assess personal dignity [58], although it has been found that relatives were more able than staff to understand when an older patient’s dignity was offended [59]. In two studies, relatives reported that staff did not sufficiently maintain the patient’s dignity, rights, and personality/identity [41, 43]. Therefore, collaboration with close family members is helpful for the complex task of maintaining residents’ identity and dignity.

When it comes to physical and sexual abuse, which is reported previously [12, 20, 21], it seems that this may be a more hidden form of abuse for relatives, since they reported it less frequently in our study. In their descriptions of the physical abuse they witnessed, relatives mainly reported co-residents as being the perpetrators. Although physical violence may be considered a serious form of abuse, relatives were not particularly explicit in their descriptions of resident-to-resident aggression. While they did describe incidences and the fear that violent episodes cause in residents, the relatives seemed more concerned about different forms of staff-to-resident abuse. Myhre et al. found that NH leaders perceived resident-to-resident aggression as a “normal part of NH life” [60]. Although relatives in our study are concerned with resident-to-resident aggression and the consequences it has on their family member, their perception of it was similar to the perception of NH leaders [60]. On the one hand, relatives tend to excuse or accept the occurrence of resident-to-resident aggression; on the other hand, they also expect staff to deal with it. This congruence of perceptions of resident-to-resident aggression between relatives and NH leaders is a problematic aspect highlighted by our study and expresses the lack of awareness of elder abuse in NH.

Our results demonstrate that all forms of elder abuse as defined by the WHO [19] are reported, but relatives reported fewer experiences with financial and sexual abuse. However, the interviews demonstrated *suspicions* of sexual abuse, and participants reported that belongings were loaned to other residents and disappeared. In our study, the latter has been categorized as psychological abuse due to the lack of respect for personal property, rather than exploitation.

### Explanations for abuse

Findings in our study revealed that relatives whose loved-one’s experienced abuse or neglect viewed low competence in staff as an explanation for elder abuse in NH, especially in managing challenges related to dementia, as well as staff unsuitable for working with these residents. These explanations may represent a proxy expression of staff burnout and work overload, known risk factors associated with elder abuse [10, 27–29]. In a study of caregivers’ concerns and experiences with neglect and abuse of NH residents, the participants believed that incidences of error, neglect and abuse were consequences of their own vulnerability, since they were not able to meet the demands of an overstrained work situation [61]. Employees’ rudeness may not be actively premeditated but rather stems from work that is extremely stressful because of understaffing, a lack of time for individualized care, interpersonal conflicts, and aggression by certain residents or their relatives [21].

These underlying explanations lead to organizational and cultural explanations for elder abuse in NH. Findings from our study revealed that relatives mostly perceived organizational factors as decisive explanations. These were factors such as low staffing, poor leadership, and a working culture characterized by fear and loyalty to employer or co-workers. According to the relative’s perception of abuse and neglect being trivialized by the NH and of poor leadership, Myhre et al. found that the occurrence of abuse was rationalized in NH, and care managers attempted to excuse why it was happening [60]. NH leaders did not have a clear perception of how they should follow up incidents of elder abuse on different levels in the organization and what their role should be in preventing elder abuse [60, 62].

Previous studies have shown that interactions resulting in abuse and neglect were more related to the care culture than being intentional by staff [63, 64]. Furthermore, relatives in our study explained that resident-to-resident aggression was caused by too few staff to look after the residents. Other studies have also connected resident-to-resident aggression to organizational factors such as staffing levels and mix and found it was largely normalized by the institutional context in which it occurred [47]. In addition, the physical environment contributed to resident-to-resident aggression [47], as it occurred in public areas such as dining rooms and hallways as well as in private areas such as a resident’s private room [65].

Elder abuse has been conceptualized as a specific form of institutional abuse within NH in earlier studies [66]. NH may be seen as a setting in which abuse and neglect occur [21] since rules and regulations in institutions, such as mealtimes and sleeping time, may be considered abusive, and the shared living space with other residents constrains individualized care. Institutional abuse may be viewed as a lack of positive response to the complex needs of residents, rigid routines, inadequate staffing, and an insufficient knowledge base within the service [67]. Additionally, the chronosystem of the ecological model of elder abuse will have an impact on norms and values, such as becoming old and no longer being considered a useful member of the society, but rather a burden. Further, factors from the societal level will affect NH institutions through ageism and budgets. Rather than supporting the notion that abuse is perpetrated by a few wicked individuals per se, one must address flaws in the system instead, with less “blame” on individuals [68]. Determinants related to abuse within institutions are complex and multifactorial, entailing various associations between personal, social and organizational factors in addition to factors within the wider society [25, 69]. This means that the risks of staff-to-resident abuse and resident-to-resident aggression extend beyond the traits and circumstances of staff who abuse or neglect the residents or the aggressive residents who harm them [25].

### Maltreatment of relatives to NH residents

In this study, the relatives’ stories demonstrated that they themselves were victims of maltreatment from the NH. Their stories comprised co-suffering with their parent’s or spouse’s suffering, rejection and ignoration from staff, lack of involvement in the resident’s situation, and staff who were directly hostile when a conflict had arisen. Relatives also felt that their cooperation occurred solely on the terms set by the NH or that they were given a role as a “troublesome” relative if they tried to interfere. Another study that explored staff-family relationships in NH had similar findings, where the relatives experienced lack of affirmation by the staff, that they were excluded from decision-making, were treated in an unfriendly manner, and gained no feedback from staff [43].

These experiences of maltreatment against relatives of NH residents have grave consequences. The relatives experienced stress and anxiety, and they were fearful of retaliation from staff towards their parent or spouse, which made relatives more inclined to accept negative NH practices. Furthermore, it made them compensate for lack of care. The key issue here is the lack of trust that emerges in the relationship between relatives and staff. A complicating factor in the description and understanding of elder abuse is that the voices of the older people themselves have generally been excluded [36]. In our study, relatives perceived themselves as important voices for their “voiceless” family member and were subsequently also worried for other residents who did not have relatives to speak for them. This perhaps speaks to the necessity of systematic, trust-based cooperation between relatives and staff. A key finding in a recent qualitative study of relatives and care staff collaboration was that staff and relatives together were able to identify factors of residents’ well-being, and family members who visited daily worked collaboratively with care staff to maintain the quality of life of their relatives and engaged in proxy decision-making. The result was that they managed to avoid abuse [45]. Including the relatives in a symmetrical collaboration on the relative’s own terms is therefore of utmost importance.

### Strengths and limitations

This is a study of relatives’ experiences with abuse and neglect of family members residing in NH and is therefore concerned with negative aspects of NH institutions. One limitation that should be acknowledged is participation bias [48]. Family members who volunteered to participate in this study were heavily involved in the issues raised by the study, as they had experienced abuse and neglect of their family member first-hand. A purposive sample was used to involve participants who were concerned more than usual about abuse and neglect in NH. In that way, they might enrich the data and thereby the knowledge about the phenomenon [50]. This study design may also result in an unbalanced representation of the reality in NH. A more balanced representation could have been made if staff and residents themselves were also interviewed. However, staff experience with abuse and neglect have been described in several studies, both quantitative and qualitative [3, 5, 11–13, 20–22, 24, 27, 30–32, 60]. Furthermore, as most residents are unable to represent experiences of abuse and neglect themselves, relatives become an important proxy, providing unique perspectives on a severe and difficult topic. It must however be stressed that they do not necessarily represent the exact experiences of the residents themselves.

All authors are registered nurses with PhD and have knowledge about NH through cooperation with NH in research and nursing student training for many years. The authors are currently working in academic professions, but also have work experience from NH. One of the authors have also worked as a NH leader. The authors have a shared interest in research regarding Quality of Care in older persons and elder abuse in various settings. This experience and knowledge provide significant contextual insight into the study phenomenon, but also entails a risk of prejudiced assumptions. We have therefore been conscious of our own preconceptions through the different steps of the research process.

In this study, we used telephone interviews due to the lockdown during the COVID-19 pandemic in the spring of 2020. During the planning phase, we perceived this as a limitation since we could not meet face-to-face and read the informants’ facial expressions and body language [50]. However, we soon discovered telephone interviews to be an advantage. The informants all conversed with us from their home environment, which had a relaxing effect on informants when telling us about challenging experiences with abuse and neglect of loved ones. This is a sensitive topic, and experiences such as abuse and neglect of loved ones may be emotionally difficult to share. However, the informants were very open-hearted, and many interviews lasted much longer than scheduled due to the many experiences the informants wished to share with us.

The interviews engaged relatives with similar experiences of elder abuse in NH from all over Norway. During the data collection, we soon discovered similarities in their stories, and the analysis further confirmed this, which increases the value of these findings to other NH where abuse and neglect may occur. The findings from our study does not necessarily describe experiences that are common for all NH residents, but rather the nature of abused NH residents as experienced by the relatives. However, it is important to stress that relatives may have a wide range of experiences with NH that are not abuse, including experiences of low-quality care, but not clearly defined as abuse, as well as the experiences of good quality care. This is not expressed through a study such as ours, which investigates the problematic aspect of elder abuse in NH institutions. In order to achieve improvement in NH quality of care and prevent abuse and neglect, problematic areas of NH practice need to be addressed.

## Conclusions

The current study concludes that relatives whose loved-one’s experienced abuse or neglect experience all forms of elder abuse in NH as defined by the WHO, and that neglect of basic care and individual rights is predominant. The relatives viewed organizational explanations as most important and had seen consequences such as depression, anxiety and aggression when their loved ones were exposed to abuse and neglect. Relatives perceive themselves as collaborators in care and are emotionally attached to their family member. Therefore, experiencing resident abuse or neglect inflicts a feeling of being mistreated themselves, particularly if the relatives are not listened to, if their notice of abuse on the part of the resident is ignored or trivialized, or if they are left with a fear of retaliation from staff towards their family member. This reveals a deep distrust among relatives directed towards NH staff and organizations. This is unfortunate, as the relatives are an important link between residents’ needs for individualized care and the nursing homes that are the ones to execute the care. Including relatives in a committed partnership with NH is not only a valuable path in the prevention of risk of abuse, but it also leads to a more sustainable healthcare with high standards of quality and safety.

## Data Availability

The datasets generated and/or analysed during the current study are not publicly available due to individual privacy could be compromised but are available from the corresponding author on reasonable request.

## Abbreviations

NFR: Research Council of Norway
NH: Nursing home
NKVTS: Norwegian Centre for Violence and Traumatic Stress Studies
NSD: Norwegian Centre for Research Data
NTNU: Norwegian University of Science and Technology
ADL: Activities of daily living
